# Predicting Training Gain for a 3 Week Period of Arm Ability Training in the Subacute Stage After Stroke

**DOI:** 10.3389/fneur.2018.00854

**Published:** 2018-10-11

**Authors:** Martin Lotze, Sybille Roschka, Martin Domin, Thomas Platz

**Affiliations:** ^1^Functional Imaging Unit, Center for Diagnostic Radiology, University of Greifswald, Greifswald, Germany; ^2^Spinal Cord Injury Unit, Centre for Neurorehabilitation, Intensive and Ventilation Care, BDH-Klinik Greifswald, University of Greifswald, Greifswald, Germany

**Keywords:** subacute stroke, pyramidal tract integrity, upper limb motor function, arm ability training, recruitment curve steepness, diffusion weighted imaging, diffusion tractography, longitudinal

## Abstract

**Background:** Biomarkers for gains of evidence based interventions for upper limb motor training in the subacute stage following stroke have rarely been described. Information about these parameters might help to identify patients who benefit from specific interventions and to determine individually expected behavioral gains for a certain period of therapy.

**Objective:** To evaluate predictors for hand motor outcome after arm ability training in the subacute stage after stroke selected from known potentially relevant parameters (initial motor strength, structural integrity of the pyramidal tract and functional motor cortex integrity).

**Methods:** We applied the arm ability training (AAT) over 3 weeks to a subpopulation of stroke patients with mild arm paresis, i.e., in 14 patients on average 4 weeks after stroke. The following biomarkers were measured before therapy onset: grip strength on the affected hand, transcranial magnetic stimulation recruitment curve steepness over the primary motor hand area [slope ratio between the ipsilesional hemisphere (IH) and contralesional hemisphere (CH)], and diffusion weighted MRI fractional anisotropy (FA) in the posterior limb of the internal capsule (PLIC; determined as a lateralization index between IH and CH). Outcome was assessed as the AATgain (percentage improvement over training). The “Test d'Evaluation des Membres Supérieurs de Personnes Âgées” (TEMPA) was assessed before and after training to test for possible associations of AAT with activity of daily living.

**Results:** A stepwise linear regression identified the lateralization index of PLIC FA as the only significant predictor for AAT-gain (*R*^2^ = 0.519; *P* = 0.029). AAT-gain was positively associated (*r* = 0.59; *P* = 0.028) with improvement in arm function during daily activities (TEMPA).

**Conclusions:** While all mildly affected patients achieved a clinically relevant therapeutic effect, pyramidal tract integrity nevertheless had a modifying role for clinical benefit.

## Introduction

Hand motor outcome is one of the clinically most important parameters after stroke and about half of stroke survivors remain to be significantly delayed in distal pinch grip performance (1) 3 months after stroke. In order to understand mechanisms of motor recovery processes for evidence based interventions the identification of parameters able to predict motor gain during training is an important strategy. This might help to identify patients who are responding best for a given therapy and is an important step on the way for individualized therapy planning using biomarkers. It has been suggested before (2), that predictive parameters for upper limb outcome after stroke might be related to three aspects of motor system integrity: the initial motor performance (e.g., motor score), the functional integrity of the motor system [e.g., quantified by motor recruitment of hand muscles, indicated with motor evoked potential (MEP) amplitude height using transcranial magnetic stimulation (TMS)], and the structural integrity of the motor system [for white matter connectivity for instance the fractional anisotropy (FA) of the pyramidal tract at the height of the posterior limb of the internal capsule (PLIC)]. All these aspects contribute to the PREP1 algorithm (Predict Recovery Potential; Version 1) suggested for upper limb outcome prognosis from the subacute stage after stroke (3).

With respect to the motor outcome used for prediction, it is important to consider that those patients who are left with good motor performance initially are those who are leaving therapy near to normal (4, 5). Contrarily, those patients who are strongly impaired initially have a wider range for motor gain, i.e., no ceiling effect. Compared to the level of performance after training, gain might be better suited to indicate benefit by a given therapy approach in damaged patients. In addition, training gain directly expresses the effect of a specific training and according to the proportional recovery model it is likely to detect training effects among both more and less severely affected patients (6).

Secondly, TMS induced MEP amplitude is a clinically valid method for predicting motor outcome in patients after stroke (3, 7). The recruitment of motor assemblies in an increasing stimulus intensity protocol (recruitment curve steepness) and its ratio between the affected and the non-affected hemisphere has been described to be a valuable monitor for corticospinal integrity (8, 9).

Thirdly, PLIC FA has been reported in several studies to show a predictive value for motor impairment for the acute (10) to subacute (between 1 week and 3 months after onset) stage after stroke [for a recent review see (11)]. An earlier study determined the relevance of PLIC FA for Fugl-Meyer score gain after a 3 week of robotic therapy upper limb intervention (12). Riley et al. reported relevance of PLIC FA only of the part interconnected with the primary motor cortex (M1) and the dorsal premotor cortex (dPMC). Other compartments such as those interconnecting the supplementary motor area (SMA) or the ventral premotor cortex (vPMC) showed no relevance for impairment outcome.

We measured these three biomarkers before an impairment-oriented training (IOT) during inpatient rehabilitation therapy in the subacute stage after stroke on a number of stroke survivors with mild hand motor affection.

For IOT we applied the arm ability training (AAT), an evidence based training recommended for stroke patients with mild to moderate upper limb impairment and dexterity deficits (13). AAT was performed for a period of 3 weeks in the subacute stage after stroke (4 weeks on average). The predictive power of the three biomarkers for AAT gain was assessed using a stepwise linear regression. In order to assess the relevance of gain with training tasks for arm use during daily activities we correlated AAT-gain with those scored with the TEMPA, a timed measure of (non-trained) tasks resembling daily life activities (14).

## Methods

### Participants

**Figure 1:**
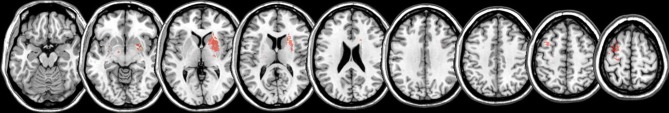
Lesion map. Lesion mapping (color coded in red) overlay on the MNI reference brain for all 14 patients investigated. Predominantly right subcortical and left hemispheric cortical lesions are seen.

Overall 19 patients were recruited from the BDH Neurorehabilitation Center in Greifswald. All patients had been diagnosed with a first-ever unilateral supratentorial anterior circulation ischemic stroke. To be eligible for the motor training patients had to be able to grasp smaller objects and to move their arm against gravity [with a score of ≥3 at the Medical Research Council (MRC) scale for pinch grip and ≥4 for shoulder abduction and elbow flexion respectively with notable impairment (15)]. Other inclusion criteria were: (1) first ischemic supratentorial anterior circulation stroke, (2) unilateral upper limb impairment (3) no contraindications for MRI and TMS (e.g., ferromagnetic devices, epilepsy) (4) older than 18 years, (5) 2 weeks to 4 months after stroke, (6) no other neurological or psychiatric diseases, (7) no current pregnancy, (8) being able to consent for study participation (e.g., no severe cognitive impairment). Five patients initially recruited did not complete the study procedures and were drop-outs. Reasons for drop-out had been: inpatient rehabilitation therapy was not longer covered by health insurance, an additional stroke occurred, agoraphobia in the scanner tube, and lack of compliance. Complete data sets of 14 patients were included in the analysis (11 were male, 3 female; age 59.71 ± 12.10 years; range = 33–74 years; 12 right-handed; average score of handedness 82.6 ± 33.72 according to the Edinburg handedness inventory (16), Mini Mental Status (MMS) averaged on 27.00 ± 2.32, National Institutes of Health Stroke Scale [NIHSS (17)] with 2.64 ± 1.78, and lesion size was 7.09 ± 15.15 ccl (see lesion map on Figure 1, Table 1). With respect to initial motor impairment the Motricity Index (MI) Upper Extremity Score (17) ranged with 77–92 and the NIH stroke scale (NIHSS) ranged with 0–6, both indicating mild to moderate impairment. In addition, the REsistance to PAssive movement Scale [REPAS, arm score (18)] indicated mostly no spasticity (range 0–2). Patients were included 2 to 9 weeks after stroke (on average 4.61 ± 1.93 weeks).

**Table 1:** Demographic and clinical data of stroke patients.

**Subject**	**Age**	**Gender (male/female)**	**Time since stroke (weeks)**	**Lesion hemis-phere**	**Lesion location^a^ and volume (ccl)**	**MMST^b^**	**REPAS^c^**	**NIHSS^d^**	**MI^e^**
1	65	m	2	Left	sc; put (0.2)	30	2	2	79
2	57	m	5	Left	sc; pt; ic (0.3)	27	1	1	92
3	45	m	5	Right	sc; pt; ic (0.6)	30	0	2	85
4	73	m	6	Left	c; M1; S1; parieto-temporal (57.5)	24	1	5	85
5	52	f	4	Left	sc; pt (1.2)	23	0	5	84
6	54	m	2.5	Left	sc; ec (0.3)	28	0	1	77
7	73	m	6	Left	sc; pt (3.4)	27	2	0	85
8	73	f	4.5	Left	sc, pt (1.0)	23	0	2	77
9	52	f	3	Right	sc; ic; put (11.0)	27	0	1	84
10	62	m	3	Left	sc, pt;(0.8)	28	0	6	84
11	74	m	4.5	Right	sc; pt; ic; put (14.4)	27	0	4	77
12	33	m	9	Left	sc; pt; ic (0.9)	30	0	3	77
13	57	m	3	Left	sc; pt; ic; put (6.1)	27	2	2	85
14	66	m	7	Right	sc; pt; ic; ec; put (2.2)	27	0	3	77

a lesion location: sc, subcortical; c, cortical; ic, internal capsule; pt, pyramidal tract; ec, external capsule; M1, primary motor cortex; S1, primary sensorimotor cortex; put, putamen.

b MMST, mini mental status test.

c REPAS, resistance to passive movement scale, affected arm.

d NIHSS, National institutes of health stroke scale.

e *MI, Motricity index*.

Participants provided written informed consent and the study was approved by the Ethics Committee of the University Medicine Greifswald (BB51/07a).

### Outcome measure

We selected increase in AAT performance for the affected upper limb as measure of training gain. Duration (seconds) of performance pre minus post was calculated, averaged over all 8 AAT tasks, and expressed individually as percent performance increase.

### Associations of outcome measure gain with changes in activities of daily living (ADL)

Transfer to non-trained tasks involving affected upper extremity arm use resembling activities of daily living was assessed as improvement in the TEMPA time score (14), documented before and after training.

### General study design

**Figure 2:**
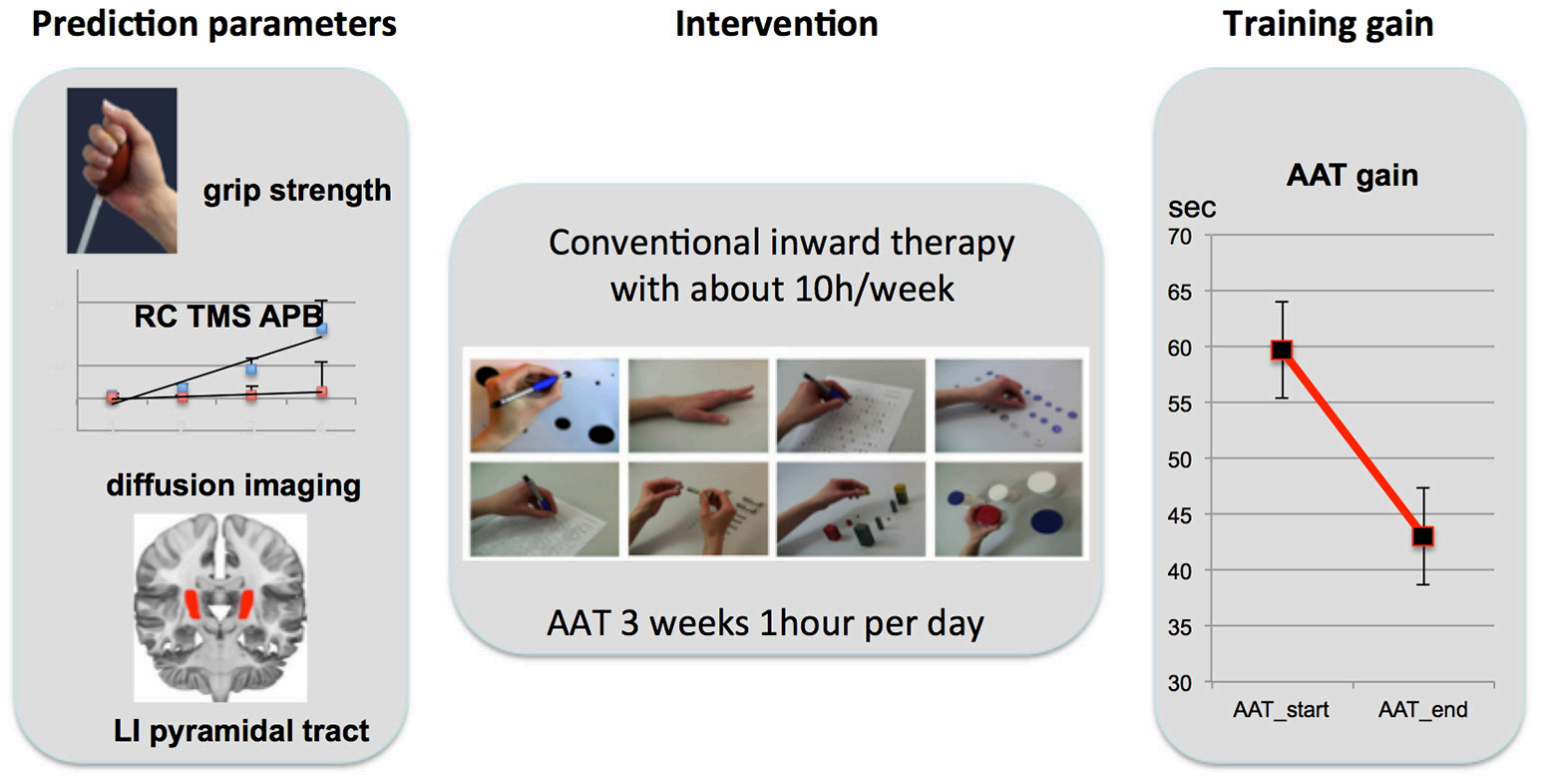
Study Design. **Left**: Prediction parameters were selected from three different levels of motor integrity: motor function (grip strength), motor cortex neurophysiology [ratio of RC slope between the IH (red) and CH (blue); schematic plot of all participants; standard error indicated with lines over average plots; APB, abductor pollicis brevis], and structural integrity of the pyramidal tract (PT; lateralization index of fractional isotropy: LI_FA_). Intervention (AAT; **middle**) was highly standardized in this trial with 1 h 5 days a week over 3 weeks. After 3 weeks of training motor gain was assessed with percentage improvement (**right**: decrease in execution time averaged over all 8 AAT tasks).

Patients were preselected by SR within the first days when they entered the rehabilitation hospital. Our study occupational therapist (SR) and one of the neurologists (TP or ML) visited the patients for suitability for AAT training. When the patient consented to participate predictive parameters were assessed and intervention was started within 3 days. Intervention was provided for 3 weeks and post training measurement was completed within less than 3 days of training completion. Figure 2 provides an overview on the study design.

### Motor training

Inpatient rehabilitation therapy was individualized to account for individual therapeutic goals for different domains (cognition, speech and language, arm rehabilitation, rehabilitation of stance, balance and gait, and psychological counseling). Overall, therapy amounted on average for 10 h therapy/week. In addition, all participants received the Arm Ability Training (AAT) 1 h per weekday for 3 weeks. This repetitive and standardized training targets different sensorimotor abilities such as fast finger movements, arm-hand steadiness, aiming, visuomotor tracking, and dexterity of the affected arm and hand (13). The AAT has been shown to be an effective training for mild to moderate arm paresis after stroke (18, 19). Training comprised eight different tasks for the affected arm and hand: aiming, tapping, crossing circles, turning coins, labyrinth, nuts and bolts, placing small objects, placing large objects. The tasks were repetitively trained in blocks; four blocks for each task, each lasting approximately 1 min. At the first day of training, the individual number of repetitions within 1 min for each task and block was determined for every patient based on the patient's individual motor capacities and kept constant for the following training days. Time needed for the execution of each of the eight trained tasks was recorded daily by the therapist (SR). Improved performance was indicated by reduced execution time keeping accuracy demands of the tasks constant. For the pre assessment before training we averaged the first two AAT-testing runs for each subtest. For the post evaluation we averaged the last 2 measurements of day 15 for each subtest. Performance feedback was given verbally and visually as intermittent knowledge of result in order to maintain motivation.

### MRI data acquisition

We used a 3T MRI-scanner (Verio, Siemens, Erlangen, Germany) with a 32 channel head coil. T1-weighted imaging for lesion mapping was carried out using a sagittal 3D MPRAGE with 176 slices, a spatial resolution of 0.98 × 0.98 × 1 mm^3^. The field of view was 250 × 250 mm^2^ corresponding to an acquisition matrix of 256 × 256. Repetition time was 1690 ms, echo time 2.52 ms, total acquisition time 3:50 min. In both sequences GRAPPA with a PAT factor of 2 was used. In addition, we applied a Siemens MDDW (Multi Directional Diffusion Weighting) sequence with the following parameter setup: voxel size: 1.8 × 1.8 × 2.3 mm^3^, 55 slices, 1 acquisition and 64 directions. One b0-volume was measured and *b* = 1000 s/mm^2^ was used for the diffusion-weighted images. TR was 10500 ms, TE: 107 ms and the total scan time was 12 min. No acquisition matrix interpolation was used.

### MRI-data evaluation

#### Lesion volumes

Lesion volumes were calculated by manually drawing the border of the lesion in the high-spatial-resolution T1-weighted image for each slice and by calculating the resulting volume (cc) with MRIcro (http://www.mccauslandcenter.sc.edu/crnl/mricro). Overlay of ROIs was visualized using Non Parametric Mapping (NPM; Chris Rorden; Vers. 2013).

#### DWI data evaluation

After conversion of the MDDW diffusion data to the NIFTI format, the FSL (v5.0.6) tool EDDY_CORRECT was used to correct for eddy-current and motion-related artifacts, including an appropriate correction of the diffusion gradient vector table. One participant (patient 14) had to be excluded from further DTI-analysis because of movement artifacts. The FSL-tool DTIFIT was used to calculate the diffusion tensor as well as related measures such as fractional anisotropy. Additionally, the individual T1 images were coregistered to their respective DWI data and, after skull stripping, (non-) linearly transformed to MNI space using FSL FLIRT and FNIRT. The combined inverse of the final non-linear transformation (DWI->T1->MNI) was created, allowing for a reverse-normalization of MNI space atlases or regions-of-interest into the individual subject space (20).

The binary ROIs of the pyramidal tract (posterior limb of the internal capsule, PLIC) [JHU Whitematter Label Atlas, (21, 22)] were transformed from MNI space into subject space using the aforementioned inverse transformation and for each ROI the mean FA values were extracted.

The lateralization index for the fractional anisotropy between the ipsilesional and contralesional hemisphere were calculated as suggested before [(LI = FA_IH_-FA_CH_)/(FA_IH_+FA_CH_); (9, 23, 24)].

FSL PROBTRACKX was used to differentiate compartments in the PLIC deriving from different seeds. Five cortical target regions were selected: vPMC [sphere of 10 mm around the cluster (MNI-coordinates: −48, 3, 21) showing an fMRI-increase over AAT in the same participants (25)], the dPMC, M1, S1were chosen from the Human Motor Area Template (26) and superior parietal lobe (SPL) from the Anatomy toolbox for SPM (27).

#### TMS-measurement

Each participant's T1- MRI and head and brain surface models were used for stereotaxic co-registration of the participant's brain with the TMS coil. This enabled online neuroanatomic control of coil positioning during TMS assessment. Patients were seated in a reclining chair and instructed to remain relaxed. Surface electromyography (EMG) from participants' abductor pollicis brevis (APB) muscle was monitored using the motor evoked potential unit of (Dantec Keypoint® by Alpine Biomed ApS, Skovlunde, DK). Application of TMS was performed with a 75 mm figure-8 passively cooled coil (MCF-B65) and the MagPro X100 Magnetic Stimulator and (MagVenture A/S, Farum, DK). The TMS coil was oriented tangentially to the scalp with the handle pointing back and away from midline at 45° during stimulation of both primary motor cortices (M1).

The latency and amplitude of the M -waves were used as measures of α motoneuron excitability. M waves and MEPs were recorded from silver chloride surface electrodes overlying the APB muscle of each hand. M-waves were elicited using supramaximal electrical stimulation of the median nerve at the wrist. The resting motor threshold (RMT) and recruitment curves (RCs) were used as measures of corticomotor excitability. Relaxation was monitored by visual feedback of the EMG signal within sweeps of 100 ms from stimulus onset. After amplification and band-pass filtering (2 Hz to 10 kHz) the EMG signal was digitized and stored for off-line analysis. The RMT for each hand was defined as the minimum stimulus intensity that produced MEPs > 50 μV in at least 5 of 10 consecutive trials. RCs were derived from the MEP amplitude obtained at 90, 110, 130, and 150% of RMTs, from 8 valid stimuli per intensity. Individual trials were examined, and any traces showing voluntary EMG activity or artifacts were discarded. The MEP amplitude was measured peak to peak from the average of 8 valid trials. MEP amplitudes for the RC were normalized to M-wave amplitude. MEP-amplitudes for the recruitment curves (RC) were divided by M-Wave-amplitude for each hand to provide a corrected MEP recruitment curve from the affected hemisphere of each patient [method adopted from Ward et al. (8)]. The slope of RC was determined from the line of best fit using least squares. We used the proportional relation (RC slope ratio) between the affected and the unaffected hemisphere, because we intended to express the decrease of motor recruitment instead of absolute values [again adopted from Ward et al. (8)].

## Statistical approach

All tests were performed with SPSS (Statistical Package for the Social Sciences; PASW-Statistics Version 21). Bilaterally assessed motor scores were tested for differences between affected and unaffected hand. As REPAS and MI are not interval scaled, these were tested with Wilcoxon paired tests, grip strength was tested using a pairwise *t*-test.

Associations of training gain were assessed with percentage AAT improvement and change scores of time needed to perform the different TEMPA tasks using Pearson correlation. Both scores were tested for significant differences between pre and post easurements using a paired *t*-test (AAT) or Wilcoxon paired *t*-tests (TEMPA affected and unaffected hand).

We tested for effects between the four TMS-measurements of recruitment curve steepness using a two by two rmANOVA with the factors TIME (pre, post) and SIDE (ipsilesional, contralesional).

For prediction of training gain (percentage AAT improvement) we first calculated Pearson correlations between each possible predictor and training gain. This analysis was restricted to those patients with complete assessments for all three aspects of motor integrity (grip strength, TMS-steepness ratio, LI_FA_ PLIC). In addition, these variables were entered into a stepwise linear regression analysis (probability to enter in the model *p* = 0.05; probability to leave the model *p* = 0.10) to determine an overall prediction model for training gain.

Furthermore, DTI-FA values for the differentiated PLIC compartments, i.e., connected to dPMC, vPMC, M1, S1, and superior parietal cortex were compared between the hemispheres using paired *t*-tests.

## Results

### Motor performance

**Figure 3:**
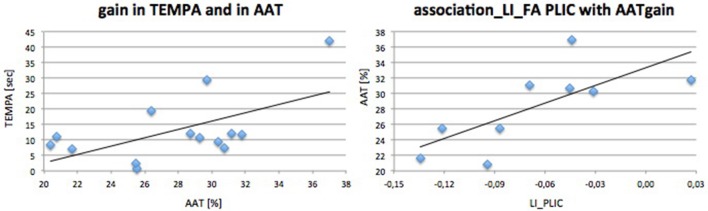
Performance gain associations. Associations and linear regression plot of trained (AAT gain) and ADL (TEMPA)-motor score gain (**left**) and AAT gain with DTI FA PLIC (**right**).

Initially we found a marked difference in all motor parameters between the affected and unaffected hand: grip strength [*t*_(13)_ = 5.72; *p* < 0.001], REPAS (*z* = 2.25; *p* = 0.024) and MI (*z* = 3.74; *p* < 0.001). Arm ability training showed an overall average gain of 27.79 ± 4.70% from day 2 to day 15 which represents a relevant gain over time [*t*_(13)_ = 20.07; *p* < 0.001]. The time needed to perform the TEMPA tasks improved over time for the affected side (13.16 s; *z* = 3.30; *p* = 0.001) but not for the unaffected (1.96 s; *z* = 1.85; n.s.). Improvement in AAT and TEMPA for the affected upper limb were positively associated (*r* = 0.59; *p* = 0.028; see Figure 3).

### TMS-parameters

When testing for changes over time and differences between sides for the variable RC-steepness we found no significant effect, neither for time (*F* = 0.33; *p* = 0.58), nor for side (*F* = 2.10; *P* = 0.18), and no interaction (time ^*^ side: *F* = 3.64; *P* = 0.089).

### Diffusion tractography

FA-values between pyramidal tracts as measured at the height of the internal capsule/posterior limb differed significantly, i.e., were lower on the affected side (ipsilesional hemisphere, IH: 0.59 ± 0.066; contralesional hemisphere, CH: 0.68 ± 0.032; *t*_(12)_ = 4.59; *p* = 0.001). Variability of PLIC FA-values was larger in the IH (SD = 0.066) than in the CH (SD = 0.032) illustrating the impact of lesion on PLIC FA.

**Figure 4:**
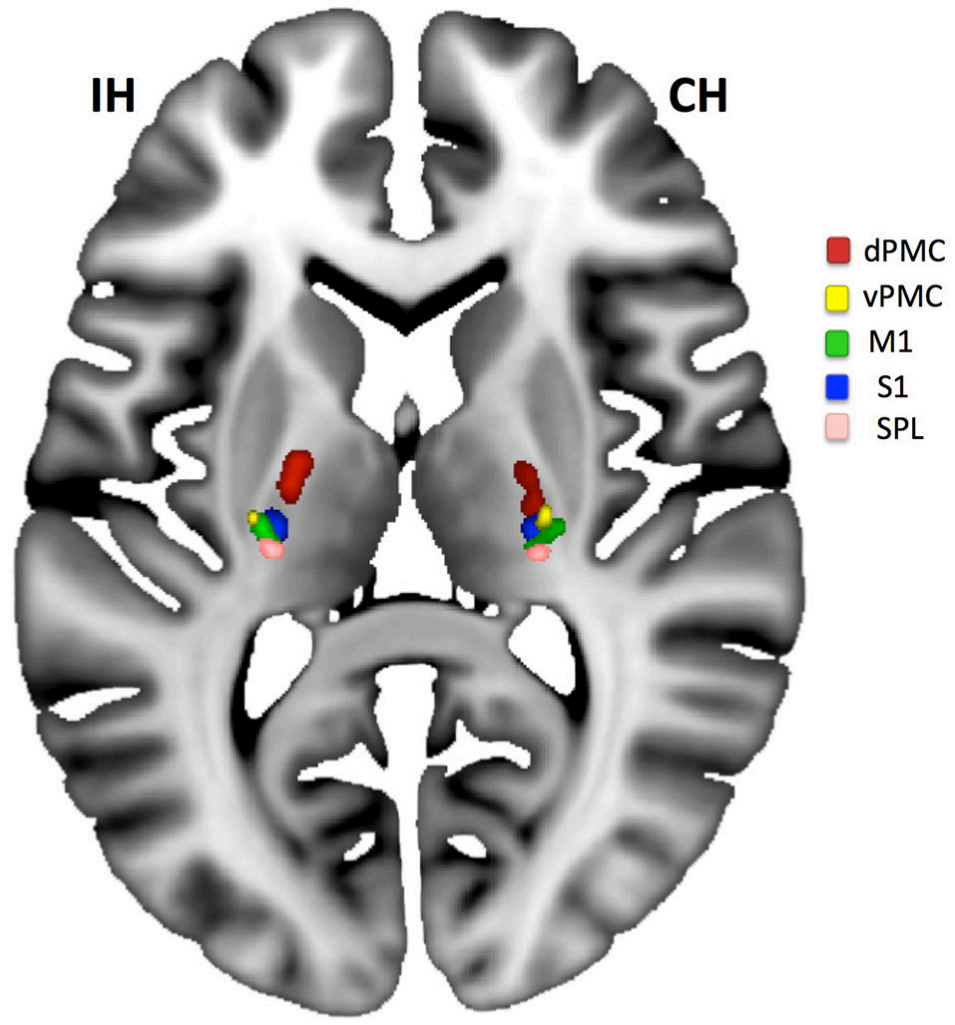
Compartments of the PLIC. Compartments of PLIC calculated for the ipsilesional (IH) and contralesional (CH) hemisphere for all patients and overlaid on the MNI-reference brain. An area on the IH with lesion caused tract loss can be located between dPMC (red) and M1 (green)/S1 (blue). Only dPMC and M1 (if corrected for 5 comparisons) showed a significant decrease in FA when IH was compared to CH.

When differentiating the pyramidal tract into subunits from five different motor regions such as dPMC, vPMC, M1, S1, and superior parietal cortex (see Figure 4) we found that only the compartments from dPMC and M1 differed between hemispheres regarding their FA values [dPMC: *t*_(13)_ = 4.58; *p*_*c*_ = 0.005; M1: *t*_(13)_ = 3.39; *p*_*c*_ = 0.025]. When testing for similarity of variances using a Levene Test (correcting for 5 comparisons) we found larger variances for the tract from the M1-seed (*F* = 9.42; pcorr = 0.025) and the tract from the vPMC-seed (*F* = 11.06; pcorr = 0.015) for the ipsilesional hemisphere compared to the contralesional.

#### Correlation of initial biomarkers with motor outcome after treatment

Initial motor performance (grip strength) was negatively associated with AAT gain (*r* = −0.68; *p* = 0.021) indicating that those who were most impaired in hand flexion strength progressed best during training. LI_FA_ PLIC was positively associated with AAT gain (*r* = 0.80; *p* = 0.005). TMS steepness ratio was not associated with AAT gain. Outcome parameter and prediction parameters were independent of age or time since stroke.

#### Stepwise linear regression analysis

FA asymmetry of the pyramidal tract was the only significant predictive factor for the primary outcome parameter (AAT gain: *R*^2^ = 0.519; *t* = 2.75; *p* = 0.029, β = 0.72). Other factors (grip strength, TMS RC ratio) had no additional predictive value for training gain. No predictive value was observed when using the FA of each single compartment of the PLIC as a predictor for AAT gain.

## Discussion

Our study identified that the pyramidal tract integrity was predictive for arm ability training gain for individuals in the subacute stage after stroke with a mildly impaired upper limb. Other factors tested had no relevant predictive effect on the primary outcome parameter. Training gain was positively associated with improvements observed with the TEMPA, a test resembling activities of daily living performed indicating a good generalization of recovered upper limb function by AAT to non-trained arm activities.

The data raises the question whether a lack of pyramidal tract integrity limits therapeutic achievement in absolute terms or would necessitate a longer span of therapy for a comparable achievement of high arm and hand motor function.

Other groups already reported the importance of pyramidal tract integrity for upper limb motor outcome in the subacute stage after stroke (7, 28). The asymmetry of fractional anisotropy within the posterior limb of the internal capsule expressed as a lateralization index (LI) is a robust quantitative parameter for assessing the intactness of the cortico-spinal tract (28). Tracts from the dorsal (dPMC) and ventral (vPMC) premotor cortex, the supplementary motor area (SMA), M1 and primary somatosensory cortex (S1), and superior parietal cortex are passing through this structure (29) enabling a global assessment of asymmetry in patients with only small lesions of the pyramidal tract. Our study extended the importance of PLIC integrity for patients in a subacute stage with only mild impediment of the upper limb function.

We applied a highly standardized, clinically effective, comprehensive and repetitive arm training over a period of 3 weeks (18, 19). It is noteworthy that we observed substantial behavioral gains across a set of different sensorimotor (AAT) tasks that involve different abilities with different learning dynamics (30) and different cortical network nodes (31). And yet, for all these tasks that showed parallel improvement over the course of 3 weeks in these stroke patients, the integrity of the efferent pathway determined about 50% (*R*^2^ = 0.519) of the magnitude of recovery. While cortico-subcortical networks (32) are critically involved in learning induced by the AAT, the intactness of the corticospinal tract is highly relevant for any improvement of sensorimotor efficiency (33).

The impairment of primarily components of M1 and dPMC of the PLIC [see also (34)] was verified by a probabilistic differentiation of pyramidal tract compartments: only these two compartments showed relevant ipsilesional decrease. However, for all five compartments, the ipsilesional hemisphere descriptively showed a larger variance in FA-values indicating the impact of pathology on the FA-values which was absent for the contralesional hemisphere.

The moderate lesion load was due to our patient selection with inclusion of subjects with mild upper extremity paresis who are known to benefit from the AAT. In addition we applied the FA-PLIC asymmetry since this measure has been solidly proven before to be associated with upper limb motor outcome (9, 24). In consideration of the patients selection (optimal for AAT therapy), the lesions of patients were diverse (see Figure 1). This might well be the reason for no predictive value of a single compartment of the PLIC (see Figure 4) but an overall effect in stepwise linear regression for AAT gain instead.

In a recent meta-analysis the authors concluded that over different TMS-studies the affected hemisphere showed higher MEP-thresholds than the unaffected hemisphere or those observed in healthy controls (HC). In contrast, the unaffected hemisphere was not different in MEP compared to HC (35). We here used a lateralization index as suggested before (8, 9) and corrected for peripheral pathology (M-wave) to focus on central pathology following stroke. In spite of these normalization and correction processes TMS-parameters indicating M1 functional integrity showed no predictive value for therapeutic gain in our setting.

Although initial grip strength showed a negative association with AAT gain, indicating that those who are starting training with more strength impairment have more capacity to improve comprehensive motor function over time, it had no predictive value for AAT gain in stepwise linear regression. Strength of the affected hand has been demonstrated to be a valuable predictor in many upper limb motor outcome studies for the subacute stage after unilateral stroke [for extension (3); for flexion (36)]. However, Xu et al. (37) demonstrated that whereas strength predominantly recovers in the first 4 weeks after stroke other parameters such as independent movements of fingers, essential for precise movements as trained in the AAT, are recovering constantly over a longer period of about 3 months. Given the fact that our patients were included after 4.61 weeks after stroke on average, recovery of strength might have taken place already after inclusion of patients. This might well had an impact on the sensitivity of strength as a predictive measure.

Another explanation for our results might be that grip strength and PLIC integrity are both measures related to corticospinal tract integrity and that PLIC integrity had been the measure more directly linked to our outcome as also indicated by the univariate analyses. This again might be related to the fact that our measure of PLIC integrity integrated other relevant aspects such as dPMC connectivity in addition to M1 connectivity. Similarly, while M1 is crucial for motor learning and has its role with sensorimotor learning during the AAT (31), cortico-subcortical connectivity seemed to be more relevant as modifier of clinical benefit by the training, again presumably by its relevance for sensorimotor integration and learning (32).

The present work has some limitations. The usage of stepwise regression is suboptimal for the evaluation of biomarkers in small samples. Larger samples might be more sensitive for investigating the predictive value of TMS-parameters for motor gain associated with effective motor training for upper limb impairment after stroke. In addition, without a control group with no AAT we cannot conclude that changes observed were caused by the special impairment oriented training procedure. Subscore analysis of the eight different movement types trained in the AAT might differentiate even better who is profiting from which subtype of AAT. However, the small sample size did not allow for a further differentiation of analysis in subtests. The total AAT gain does, however, resemble an overall improvement in sensorimotor efficiency (30) which can be considered an important and clinically relevant information.

## Conclusion

In a subgroup of stroke patients with mild arm paresis we demonstrated the predictive relevance of fractional anisotropy lateralization of the PLIC for the gain in a 3 week arm ability training. The training improved upper limb function on average by 27% and showed a significant positive association with improvement in arm function during daily activities. Especially for the early phase of training (first days) sensorimotor integration is extremely important for achieving gain in motor training (31, 32, 38). In addition, dPMC and vPMC are especially important for gaining motor recovery after subcortical stroke [for dPMC (39, 40); for vPMC (25)]. A decrease of affected hemisphere pyramidal tract FA is therefore associated with impairment in sensorimotor integration hampering relearning of motor function especially in the early phase of training. In view of the limited number of subjects our data need to be interpreted cautiously. More longitudinal studies on evidence based interventions are needed with larger patient cohorts to not only understand the most robust predictors for arm ability outcome but also to find strategies for objective therapy decisions for individuals with motor impairment in an early stage after stroke.

## Ethics statement

This study was carried out in accordance with the recommendations of the International Neuroscience Community and the German Community for Neurology. The protocol was approved by the Ethics Board of the University Medicine, Greifswald. All subjects gave written informed consent in accordance with the Declaration of Helsinki.

## Author contributions

ML and TP designed and supervised the study. SR performed the patient selection and training. MD and ML evaluated the data. All authors wrote the manuscript.

### Conflict of interest statement

The authors declare that the research was conducted in the absence of any commercial or financial relationships that could be construed as a potential conflict of interest. The reviewer LD and handling Editor declared their shared affiliation.
